# A fast, efficient chromatin immunoprecipitation method for studying protein-DNA binding in *Arabidopsis* mesophyll protoplasts

**DOI:** 10.1186/s13007-017-0192-4

**Published:** 2017-05-22

**Authors:** Jeong Hwan Lee, Suhyun Jin, Sun Young Kim, Wanhui Kim, Ji Hoon Ahn

**Affiliations:** 1 Department of Life Sciences, Korea University, 145 Anam-ro, Seongbuk-gu, Seoul, 02841 Republic of Korea; 2 Department of Life Sciences, Chonbuk National University, 567 Baekje-daero, deokjin-gu, Jeonju, Jeollabuk-do 54896 Republic of Korea

**Keywords:** *Arabidopsis*, Chromatin immunoprecipitation, Protoplasts, Transcription factor, Transient expression

## Abstract

**Background:**

Binding of transcription factors to their target sequences is a primary step in the regulation of gene expression and largely determines gene regulatory networks. Chromatin immunoprecipitation (ChIP) is an indispensable tool used to investigate the binding of DNA-binding proteins (e.g., transcription factors) to their target sequences in vivo. ChIP assays require specific antibodies that recognize endogenous target transcription factors; however, in most cases, such specific antibodies are unavailable. To overcome this problem, many ChIP assays use transgenic plants that express epitope-tagged transcription factors and immunoprecipitate the protein with a tag-specific antibody. However, generating transgenic plants that stably express epitope-tagged proteins is difficult and time-consuming.

**Results:**

Here, we present a rapid, efficient ChIP protocol using transient expression in *Arabidopsis* mesophyll protoplasts that can be completed in 4 days. We provide optimized experimental conditions, including the amount of transfected DNA and the number of protoplasts. We also show that the efficiency of our ChIP protocol using protoplasts is comparable to that obtained using transgenic *Arabidopsis* plants. We propose that our ChIP method can be used to analyze in vivo interactions between tissue-specific transcription factors and their target sequences, to test the effect of genotype on the binding of a transcription factor within a protein complex to its target sequences, and to measure temperature-dependent binding of a transcription factor to its target sequence.

**Conclusions:**

The rapid and simple nature of our ChIP assay using *Arabidopsis* mesophyll protoplasts facilitates the investigation of in vivo interactions between transcription factors and their target genes.

**Electronic supplementary material:**

The online version of this article (doi:10.1186/s13007-017-0192-4) contains supplementary material, which is available to authorized users.

## Background

Gene expression is a primary step that connects genotype and phenotype, and transcriptional regulation by transcription factors is considered an important determinant of phenotype [1]. Unraveling the molecular mechanisms underlying the regulation of gene expression is thus pivotal to understanding how genotype is translated into phenotype in living organisms. Transcription factors, sequence-specific DNA-binding proteins, bind to specific DNA sequences of their target genes to regulate gene expression. To study the interaction between transcription factors and their target sequences, a number of in vitro methods have been developed, such as electrophoretic mobility shift assays (EMSA) [2] and DNA–protein-interaction enzyme-linked immunosorbent assay (DPI-ELISA) [3, 4]. However, such methods generally have limited utility because the assays do not occur within the context of the cell. Recently, a microarray-based method such as protein microarray has been developed and has facilitated the identification and characterization of target genes that are bound by a specific transcription factor [5, 6]. This microarray-based approach also has some limitations, including very high background signal, a low dynamic range of expression levels, and a large amount of total RNA required for quantification [7], as the microarray technique is based on hybridization. Furthermore, several factors such as microarray surface chemistry, length and position of oligonucleotides, and quality of the proteins affect accuracy and reproducibility of protein microarray technology.

Chromatin immunoprecipitation (ChIP) is a powerful tool for the investigation of interactions between DNA-binding proteins and genomic DNA in vivo [8]. ChIP assays can be coupled with microarray (ChIP-chip) or deep sequencing (ChIP-seq) for genome-wide analyses. These combined ChIP analyses provide important information about DNA-binding motifs and putative target genes, as well as the biological roles of proteins of interest, through functional analysis of their target sequences [9–12]. In addition to its utility for the study of transcriptional regulation, ChIP can be also used to map genome-wide epigenetic modifications via the histone modifiers [13, 14].

When performing ChIP assays, chromatin-bound proteins are cross-linked, and the chromatin is sheared by sonication or nuclease treatment. Immunoprecipitation is then performed using specific antibodies to the chromatin-bound protein of interest. Thus, antibodies are one of the most important factors for a successful ChIP experiment. However, as antibodies that specifically detect an endogenous protein of interest are unavailable in many cases, transgenic plants that stably express the tagged protein of interest are used instead. This hampers the wide usage of ChIP methods for in vivo interaction studies, because generating such transgenic plants is difficult and time-consuming [15, 16].

Transient gene expression is commonly used as an alternative approach to study subcellular localization, promoter activity, and protein–protein interactions [17–19]. Among transient expression systems, plant protoplasts are frequently used [20, 21]. Plant protoplasts, as a versatile cell-based experimental system, have several advantages over other transient expression techniques such as biolistic approaches with gold particle-loaded DNA and *Agrobacterium tumefaciens*-mediated transformation of leaves. For instance, the protoplast system does not require a sterile environment, DNA transfection into protoplasts can be highly efficient, and protoplast experiments are time-efficient and cost-effective [22, 23]. The plant protoplast system can be also used for single-cell based imaging analyses such as protein localization, protein domain functions in protein targeting, and protein transporter functions in vesicle trafficking [24–27]. Thus, although the assay system using protoplasts is not considered a genuine *in planta* assay system, it is widely used to examine various intracellular signal transduction pathways involved in physiology, immunity, growth, and development [28–32].

In the past decades, many scientists have focused on the control of a single or a few genes by one or more regulators to elucidate the regulatory mechanisms underlying many cellular processes in eukaryotes. However, the results obtained from these studies are usually insufficient to explain complex developmental processes and adaptation to particular environmental conditions. Recently, integrative regulatory studies of gene regulation in animals have identified master regulators and network motifs, thereby allowing us to infer gene regulatory networks and make predictive models of gene expression [33–35]. Although integrative studies using genome-wide profiling of transcription factors are also conducted in plants [36], our current knowledge about the gene regulatory networks of transcription factors in plants remains limited, particularly considering that the *Arabidopsis thaliana* genome encodes at least 2000 transcription factors [37, 38]. Therefore, there is an increasing need for a fast and efficient ChIP method for genome-wide experiments to facilitate the study of the gene regulatory networks involved in the interaction between transcription factors and their target DNA sequences.

In this study, we report a simplified ChIP method for studying the interactions between transcription factors and their target sequences in vivo using *Arabidopsis* mesophyll protoplasts. We identify the experimental parameters affecting the transformation efficiency of ChIP assays. We also suggest that our ChIP method is suitable to examine tissue-specific, genotype-dependent, and temperature-dependent interactions between transcription factors and their target sequences in vivo. Moreover, this ChIP method can be coupled with expression profiling technologies, which can facilitate small- or large-scale analyses to investigate the molecular function of transcription factors in *Arabidopsis*.

## Methods

### Reagents

Antibodies [anti-c-Myc (Santa Cruz Biotechnology, Dallas, Texas, sc-40) and anti-HA (Santa Cruz Biotechnology, sc-7392)]

Complete protease cocktail inhibitor (Roche, cat. no. 04693159001)

Dithiothreitol (DTT) (Sigma, cat. no. D-9779)

Ethylenediaminetetraacetic acid (EDTA) (Sigma, cat. no. E-4884)

Ethylene glycol-bis(2-aminoethylether)-*N,N,N′,N′*-tetraacetic acid (EGTA) (Sigma, cat. no. E-3889)

Ethanol (Sigma, cat. no. E-7023)

Formaldehyde 37% (Sigma, cat. no. F-8775)

Glycine (Sigma, cat. no. 50046)

Glycogen (Roche, cat. no. 10901393001)

HEPES (Sigma, cat. no. H-3375)

Lithium chloride (Sigma, cat. no. L-4408)

Nonidet P-40 (NP-40) (see Comment, below)

Proteinase K (Ambion, cat. no. AM2546)

Pre-equilibrated salmon sperm DNA/protein A agarose beads (Millipore, cat. no. 16-157)

Sodium acetate (Sigma, cat. no. 127-09-3)

Sodium chloride (Sigma, cat. no. 7647-14-5)

Sodium deoxycholate (Sigma, cat. no. D-6750)

Sodium dodecyl sulfate (Sigma, cat. no. L-6026)

Tris (Sigma, cat. no. 93362)

Triton X-100 (Sigma, cat. no. T-8787)

#### Comment

NP-40 is no longer commercially available; we suggest using IGEPAL CA-630 (Sigma, cat. no. I8896) instead.

### Equipment

Bioruptor (LaboGene, Korea)

Rotator for tubes

Heat block

Eppendorf microfuge tubes (1.5 and 2 ml)

Centrifuge

Nanodrop machine (Nanodrop Technologies, USA)

Real-time PCR machine (Roche Applied Science, USA)

### Solutions

#### *1* × *PBS buffer*

Dissolve 8 g NaCl, 0.2 g KCl, 1.44 g Na_2_HPO_4_, 0.24 g KH_2_PO_4_ in 800 ml distilled water, adjust to pH 7.4 using HCl, fill up with distilled water to 1 L; [autoclave at 121 °C for 15 min and store at room temperature (20–25 °C) (RT) or 4 °C for up to 3 months].

#### *Harvest buffer*

10 mM DTT (add fresh, do not include in stock), 100 mM Tris–HCl (pH 9.4) [filter-sterilize using a 0.45-µm filter and store at 4 °C for up to 1 month].

#### *Nuclei wash buffer with Triton*

0.25% v/v Triton X-100, 10 mM EDTA, 0.5 mM EGTA, 10 mM HEPES (pH 6.5) [filter-sterilize using a 0.45-µm filter and store at 4 °C for up to 1 month].

#### *Nuclei wash buffer without Triton*

200 mM NaCl, 1 mM EDTA, 0.5 mM EGTA, 10 mM HEPES (pH 6.5) [filter-sterilize using a 0.45-µm filter and store at 4 °C for up to 1 month].

#### *Nuclei lysis buffer*

1% w/v SDS, 10 mM EDTA, 50 mM Tris–HCl (pH 8.0), 1 × protease inhibitor cocktail (make fresh each time by adding protease inhibitor cocktail just before use); [filter-sterilize using a 0.45-µm filter and store at 4 °C for up to 1 month].

#### *ChIP dilution buffer*

1% v/v Triton X-100, 2 mM EDTA, 20 mM Tris–HCl (pH 8.0), 150 mM NaCl, 1× protease inhibitor cocktail (make fresh each time by adding protease inhibitor cocktail just before use); [filter-sterilize using a 0.45-µm filter and store at 4 °C for up to 1 month].

#### *Low salt wash buffer*

0.1% w/v SDS, 1% v/v Triton X-100, 2 mM EDTA, 20 mM Tris–HCl (pH 8.0), 150 mM NaCl; [filter-sterilize using a 0.45-µm filter and store at 4 °C for up to 1 month].

#### *High salt wash buffer*

0.1% w/v SDS, 1% v/v Triton X-100, 2 mM EDTA, 20 mM Tris–HCl (pH 8.0), 500 mM NaCl; [filter-sterilize using a 0.45-µm filter and store at 4 °C for up to 1 month].

#### *LiCl wash buffer*

0.25 M LiCl, 1% v/v NP-40, 1 mM EDTA, 10 mM Tris–HCl (pH 8.0), 1% w/v sodium deoxycholate; [filter-sterilize using a 0.45-µm filter and store at 4 °C for up to 1 month].

#### *Elution buffer*

1% w/v SDS, 0.1 M NaHCO_3_. The elution buffer should be freshly prepared; [filter-sterilize using a 0.45-µm filter].

#### *TE buffer*

10 mM Tris–HCl (pH 8.0), 1 mM EDTA; [autoclave at 121 °C for 15 min and store at 4 °C for up to 3 months].

### Protocol

**Fig. 1:**
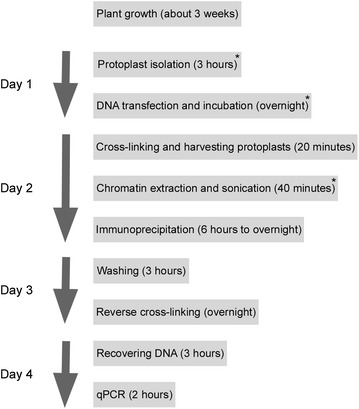
Outline of the chromatin immunoprecipitation (ChIP) protocol followed by quantitative PCR (qPCR) using *Arabidopsis* (Col-0) mesophyll protoplasts. Time required for each step is indicated in parentheses. *Asterisks* indicate some critical steps that are needed to be modified when this protocol is adapted to other plant tissues and species

The procedure for our ChIP method is outlined in Fig. 1. The ChIP protocol is optimized for *Arabidopsis* leaf tissue harvested from wild-type Columbia (Col-0) or mutants in the Col-0 background. Therefore, some modifications (for instance, protoplast isolation, the quantity of DNA and the number of protoplasts used for transfection, and chromatin extraction and sonication) may be required when this protocol is used for other plant tissues or species.

#### Arabidopsis protoplast isolation and DNA transfection

Isolate protoplasts (2 × 10^7^ protoplasts) and transfect them with DNA (40 µg) following previously described methods (see Comment, below). After isolation of protoplasts and DNA transfection, incubate protoplasts for 16–17 h at RT under continuous low light conditions (50 µmol m^−2^ s^−1^).

##### Comment

The procedures for isolation of *Arabidopsis* protoplasts and DNA transfection were previously described [21]. *Arabidopsis* plants are grown in soil at 23 °C under long-day (LD) (16 h light/8 h dark) or short-day (SD) conditions (9 h light/15 h dark) at a light intensity of 120 μmol m^−2^ s^−1^. As light is a very sensitive aspect for protoplasts and may affect the proteasome-dependent degradation of some transcription factors [39], we used low light conditions for overnight incubation (50 µmol m^−2^ s^−1^). Each ChIP experiment requires 2 × 10^7^ protoplasts (approximately 50 leaves digested in 20 ml enzyme solution) as a starting material. Before DNA transfection, the number and intactness of protoplast should be checked using the microscope and hemacytometer. Although re-assessing the number of protoplasts again after overnight incubation is not usually necessary, we recommend re-assessment of the number if inconsistent ChIP results are obtained from batch to batch. Because the tagged transcription factors for ChIP may compete with the endogenous protein to bind the target sequences, we suggest the use of protoplasts isolated from an RNA-null mutant of the transcription factor of interest. Also, the degree of expression of transcription factors and their turnover rates used in protoplast transfection could be different; it is therefore worth testing the amounts of transfected DNA and numbers of protoplasts. Furthermore, the quality of plasmid DNA or the ratio of transfected DNA and protoplast number can be scaled up or down depending on the efficiency and specificity of ChIP analyses.

#### Chromatin extraction and sonication

1. Transfer the transfected protoplasts to 2-ml tubes and centrifuge them at 1500*g* for 2 min at RT.
2. Gently remove the supernatant and wash the pellet with 1 ml of 1 × PBS buffer (pH 7.4) twice by centrifugation at 1500*g* for 2 min at RT.
3. To crosslink the proteins to the DNA, add 27 µl of 37% formaldehyde to the pellet to get a final concentration of 1% in 1 ml of 1 × PBS buffer (pH 7.4) and mix well by gently inverting the tube several times and placing it on a rotor (12 rpm) for 10 min at RT.
4. Add 2 M glycine to a final concentration of 0.1 M, and mix well by gently inverting the tube several times and placing it on a rotor (12 rpm) for 5 min at RT to quench the crosslinking reaction, and centrifuge the 2-ml tubes at 1500*g* for 5 min at 4 °C.
5. Remove the supernatant and rinse the pellet with 1 ml of ice-cold 1 × PBS buffer (pH 7.4) twice.
6. Resuspend the pellet in 1 ml of harvest buffer and mix immediately by gently tapping the tube.
7. Incubate the solution for 15 min at 30 °C and centrifuge the tubes at 1500*g* for 10 min at RT.
8. Add 1 ml of ice-cold 1 × PBS buffer (pH 7.4) to the pellet and mix immediately by gently tapping the tube.
9. Centrifuge the 2-ml tubes at 1500*g* for 5 min at RT.
10. Add 1 ml of nuclei wash buffer with Triton to the pellet and mix immediately by gently tapping the tube.
11. Centrifuge the 2-ml tubes at 1500*g* for 5 min at RT.
12. Add 1 ml of nuclei wash buffer without Triton to the pellet and mix immediately by gently tapping the tube.
13. Centrifuge the 2-ml tubes at 1500*g* for 5 min at RT.
14. Remove the supernatant and resuspend the chromatin pellet in 300 µl of ice-cold nuclei lysis buffer.
15. Resuspend the pellet by pipetting up and down with a cut-off tip (keep solution cold).
16. Take a 10 µl aliquot from the nuclei and keep it in ice. This is ‘unsheared’ chromatin. Sonicate the chromatin solution for 3–4 cycles (10 s ON and 1 min OFF for each cycle on full power using a Bioruptor). During the sonication, the tube should be placed on ice. Take a 10 µl aliquot from the chromatin solution to check the sonication efficiency.
17. Check the sonicated chromatin after reverse crosslinking (see Comment, below) and running the DNA on a 1.5% agarose gel. The DNA fragment should appear smeared from 200 to 700 bp, but major fragments should appear around 500 bp in size (refer to steps 30–35).

##### Comment

Conventional sonicators (i.e., probe type) also work well for shearing the chromatin. Time of sonication depends on the sonicator used. Sonicated chromatin samples can be flash-frozen in liquid nitrogen and stored at −80 °C for up to 3 months or can be used directly for immunoprecipitation. To reverse the crosslinking, 0.4 µl of 5 M NaCl is added to a 10 µl aliquot of the sonicated chromatin (to a final concentration of 0.2 M) and the resulting solution is incubated at 65 °C overnight. To reverse the crosslinking the chromatin-DNA complex, we did not use an SDS solution, because heat incubation at 65 °C is widely used for the process. After reverse crosslinking, go to the DNA recovery steps (31–35) to isolate the DNA.

#### Immunoprecipitation and reverse crosslinking

18. Centrifuge the 2-ml tubes at 10,000*g* for 5 min at 4 °C to pellet debris.
19. Transfer a 150 µl aliquot of the supernatant to a new 2-ml tube placed on ice and dilute tenfold with 1350 µl of ice-cold ChIP dilution buffer. Take a 150 µl aliquot from the diluted chromatin solution as the ‘10% input control’.

##### Note

The point of this step is to dilute the 1% SDS to 0.1% SDS with ChIP dilution buffer.

20. Pre-clear the diluted sonicated chromatin solution by adding 50 µl salmon sperm DNA/protein A agarose beads (use pre-equilibrated slurry) with a cut-off pipette tip for 1 h at 4 °C with gentle rotation (12 rpm).
21. Centrifuge the 2-ml tubes at 1500*g* for 3 min at 4 °C to pellet the agarose beads. Divide 400 µl aliquots of the supernatant equally into three 2-ml tubes [specific (positive) and non-specific (negative) antibody controls, and a ‘no-antibody’ (NoAb) control].
22. Add 5 µl of the appropriate antibody (1 µg) [an antibody specific to a transcription factor of interest (e.g., in our case, anti-HA antibody) into the first tube and an irrelevant antibody (e.g., anti-cMyc antibody) as a non-specific antibody control in the second tube] to the supernatant in two of the three 2-ml tubes. The third tube, to which no antibody is added, is used as a NoAb control. Incubate all the tubes at least 6 h to overnight at 4 °C with gentle rotation (12 rpm).

##### Note

The concentration of the antibody varies depending on the antibody used; check the manufacturer’s specifications.

23. Add 50 µl salmon sperm DNA/protein A agarose beads (use pre-equilibrated slurry) and continue the incubation for 1 h at 4 °C with gentle rotation (12 rpm).
24. Centrifuge the 2-ml tubes at 1500*g* for 3 min at 4 °C to pellet the mixture of agarose beads and the chromatin.
25. Wash the mixture for 10 min each time with gentle rotation (12 rpm) at 4 °C with 1 ml of the following buffers and centrifuge the 2-ml tubes at 1500*g* for 3 min at 4 °C: one time with low salt wash buffer, one time with high salt wash buffer, one time with LiCl wash buffer, and three times with TE buffer. After each wash step, remove all buffers, but be careful not to lose any beads.

##### Note

Some antibodies have a low binding affinity for the target proteins. Therefore, the stringency of the washing buffers can be varied from 150 to 500 mM salt (usually NaCl or LiCl).

26. Add 150 µl of freshly prepared elution buffer, and vortex briefly, transfer the mixture to new 1.5-ml tubes, and incubate in a heat block (65 °C) for 15 min.
27. Centrifuge the tubes at 5000*g* for 3 min at RT and carefully transfer the supernatant into a new 1.5-ml tube.
28. Repeat the elution step (step 27 and 28) three times and combine the three eluates. At the same time, add 350 µl elution buffer to 100 µl sonicated chromatin (from step 20) to serve as an input control.
29. Add 18 µl of 5 M NaCl to the eluate (to a final concentration of 0.2 M) and incubate at 65 °C overnight to reverse the crosslinking.

#### DNA recovery

30. Add 8 µl of 0.5 M EDTA (pH 8.0), 18 µl of 1 M Tris–HCl (pH 6.5), and 1 µl of 1 mg/ml proteinase K to the eluate, and incubate at 37 °C for 1 h.
31. Add an equal volume of phenol/chloroform/isoamyl alcohol (25:24:1) to each 1.5-ml tube and vortex briefly.
32. Centrifuge the tubes at 10,000*g* for 10 min at 4 °C and transfer the supernatant into a new 1.5-ml tube.
33. Precipitate the DNA with 2.5 volumes of 100% EtOH, 1/10 volume of 3 M sodium acetate (pH 5.2), and 2 µl of 20 mg/ml glycogen and incubate at −80 °C for 1 h.
34. Centrifuge the 1.5-ml tubes at 10,000*g* for 20 min at 4 °C, wash the pellets with 500 µl of 70% EtOH, and then centrifuge at 10,000*g* for 10 min at 4 °C. Dry the pellet at RT.
35. Resuspend the pellet in 30–50 µl of distilled water and store at −20 °C for up to 4 months.

#### Quantitative PCR

To assess the amount of bound target sequence, the DNA recovered from ChIP, and the 10% input DNA control, are used for qPCR. The primers used in this study are listed in Table 1. The ChIP results obtained from 3 independent biological replicates are represented as percentage of input (% Input) [40]. Chromatin immunoprecipitation experiments were carried out in two or three biological replicates (samples independently harvested on different days) with three technical triplicates each (ChIP samples processed on the same day). Error bars indicate the standard error of the mean (SEM) of two or three biological replicates.

**Table 1:** Primer sets used in this study

Gene	Name	Sequence (5′–3′)	Direction
*FT*	JH6815	GGCTATGGTTATAAGTTTCATCTTTGA	Forward
	JH6816	AATACTAACCATCCATTTGCACGA	Reverse
	JH6823	AGTTGAGATTGGTGGAGAAGACCT	Forward
	JH6824	TGATTTGGGTATCATAAAGTAAAACCA	Reverse
	JH6829	TTCAGGTTTTACTCCATCATACGG	Forward
	JH6830	TGTGATGATGTTTTTGGTCAGAGA	Reverse
*FUL*	JH6233	TCTCCGTGCATTTAACCAGA	Forward
	JH6234	TGTTGTCGAGTCCTCATTGG	Reverse
	JH6440	CAACCGAAAAGTATTGTTTTCATA	Forward
	JH6441	GCGAATTGTTGTGATCTTGC	Reverse
*GL2*	JH9192	AGCTGAAATTGGAAGGCTGAT	Forward
	JH9193	CATGGCCAGCTACAGCATTG	Reverse
	JH9194	GAGCAAACAATTGGTAGTCGGAAA	Forward
	JH9195	TGTTGTGTATCCCGGAACCAG	Reverse
*SOC1*	JH6853	CAAATCATCCATAGAAAGAGAGAGAGA	Forward
	JH6854	CAAGATGATATACTAGCGGAAATAAAA	Reverse
	JH6857	CATGAAAGCGAAGTTTGGTCA	Forward
	JH6858	GACAACAAGAGAGAAGCAGCTTTAGA	Reverse

##### Comment

For measuring the efficiency of ChIP experiments, the DNA obtained by ChIP using protoplasts isolated from a knock-out allele with transfection can also be compared with DNA obtained by ChIP using protoplasts of the knock-out allele without transfection.

## Results

### Optimal amounts of transfected DNA and numbers of protoplasts for efficient ChIP analyses using *Arabidopsis* mesophyll protoplasts

**Fig. 2:**
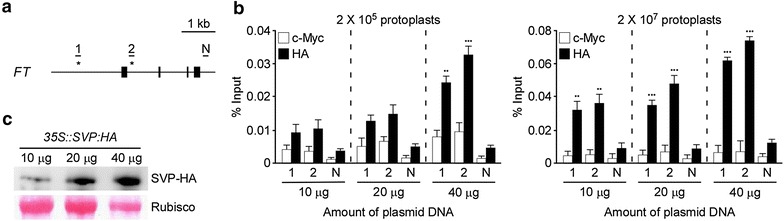
Effect of the amounts of transfected DNA and the number of protoplasts in ChIP-qPCR. **a** Diagram of the genomic region of *FT*, which contains target sequences of the SVP transcription factor. *Closed boxes* represent four exons of *FT*. The known binding sites of SVP [1 (from −1338 to −1152, relative to the translational start codon) and 2 (+159 to +416)] in *FT* are shown [17, 18]. N, a negative control (+3830 to +4068 in *FT*). **b** The effect of different amounts (10, 20, and 40 µg) of *35S::SVP:HA* DNA and the number of protoplasts (2 × 10^5^ and 2 × 10^7^) used for transfection. ChIP-qPCR assays of SVP binding to the two target sequences of *FT* are shown. The ChIP results obtained from 3 independent biological replicates are represented as percentage of input (% input). *Error bars* indicate the standard error of the mean (SEM). *Asterisks* indicate values that are significantly different from c-Myc (Student’s *t* test, **P < 0.01, ***P < 0.001). **c** SVP-HA protein expression in *Arabidopsis* protoplasts (2 × 10^7^). Anti-HA antibody was used to detect SVP-HA protein

We previously performed ChIP analyses using *Arabidopsis* (Col-0) mesophyll protoplasts to determine in vivo interactions between the SHORT VEGETATIVE PHASE (SVP) and FLOWERING LOCUS C (FLC) transcription factors and the genomic region of *FLOWERING LOCUS T* (*FT*) [17]. However, our ChIP data showed a weak correlation between direct binding of SVP and FLC with the transcriptional regulation of *FT*, suggesting that optimized conditions for transfection of DNA into protoplasts are important for ChIP analyses. To find the optimal conditions for ChIP analyses using *Arabidopsis* mesophyll protoplasts, we examined the effect of different amounts of DNA used for transfection and different numbers of *Arabidopsis* protoplasts. We transfected *35S::SVP:hemagglutinin* (*HA*) DNA in different amounts (10, 20, and 40 µg) into two sets of protoplasts (2 × 10^5^ and 2 × 10^7^). Subsequently, we performed ChIP-qPCR experiments and compared the binding of SVP protein within the *FT* sequence. The results showed that stronger binding of the SVP transcription factor within the known binding regions of *FT* was observed in 2 × 10^7^ protoplasts, compared to 2 × 10^5^ protoplasts (Fig. 2a, b). The experiment performed to test the effect of different amounts of transfected DNA showed that 40 µg of transfected *35S::SVP:HA* construct showed stronger binding than 10 and 20 µg of transfected DNA, suggesting that using more transfected DNA was effective (Fig. 2b). Furthermore, western blot analysis confirmed the expression of SVP-HA proteins in protoplasts increased with higher amounts of transfected DNA (Fig. 2c). These data suggested that using 40 µg of DNA and 2 × 10^7^ protoplasts was suitable for ChIP assays with *Arabidopsis* mesophyll protoplasts.

### ChIP analyses of three different tissue-specific transcription factors using *Arabidopsis* mesophyll protoplasts

**Fig. 3:**
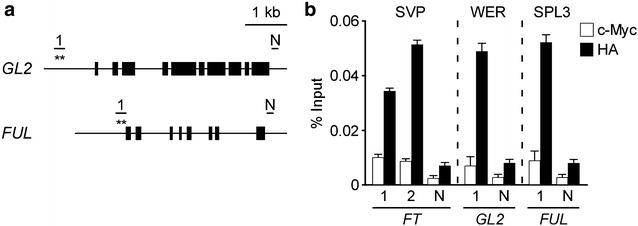
ChIP-qPCR analysis to test SVP, WER, and SPL3 binding to the genomic regions of *FT*, *GL2*, and *FUL*, respectively. **a** Diagram of the *GL2* and *FUL* genomic regions. *Closed boxes* represent exons. The known binding site of WER [1 (−933 to −889) in *GL2,* relative to the translational start codon] [44] and the known binding site of SPL3 [1 (−466 to −440) in *FUL*] [43] are shown. N is a region used for a negative control (+3774 to +3884 in *GL2*; +3322 to +3552 in *FUL*). The amplified regions within *FT* used for qPCR experiments are shown in Fig. 2a. **b** ChIP-qPCR assay of binding of SVP, WER, and SPL3 transcription factors to the genomic regions of *FT*, *GL2*, and *FUL*, respectively, using *Arabidopsis* mesophyll protoplasts. The ChIP results obtained from 3 independent biological replicates are represented as percentage of input (% input). *Error bars* indicate the SEM

In many cases, transcription factors control diverse aspects of plant growth and development in a cell type-specific manner. The SVP, WEREWOLF (WER), and SQUAMOSA PROMOTER-BINDING PROTEIN-LIKE3 (SPL3) transcription factors are specifically expressed in leaves [17], roots [41], and shoot apices [42], respectively, and the binding sites in their target genes are known [18, 43, 44]. To examine whether our ChIP method works well to test the binding of tissue-specific transcription factors to their target genes in *Arabidopsis* mesophyll protoplasts, we performed ChIP-qPCR assays by transfecting the *35S::SVP:HA*, *35S::WER:HA*, and *35S::SPL3:HA* constructs. Known binding sites of SVP (CArG motifs in *FT*), WER [(C/T)DGTT(G/A) motifs in *GLABRA2* (*GL2*)], and SPL3 [GTAC motifs in *FRUITFULL* (*FUL*)] (Fig. 3a) were amplified. A non-target region from each gene was used as a negative control (N). ChIP-qPCR analyses showed that strong binding of SVP, WER, and SPL3 transcription factors were observed in known binding regions of *FT*, *GL2*, and *FUL*, respectively (Fig. 3b), which was consistent with previous results [18, 43, 44]. No apparent binding of SVP, WER, or SPL3 was observed in negative control regions. Consistent with our data, the binding of AUXIN RESPONSE FACTOR19 (ARF19) to the *BR*-*RELATED ACYLTRANSFERASE 1* (*BAT1*) gene, which is highly expressed in vascular bundles in a tissue-specific manner, was successfully detected using *Arabidopsis* mesophyll protoplasts [45]. These data suggest that ChIP assays using *Arabidopsis* mesophyll protoplasts are useful for analysis of binding of tissue-specific transcription factors to their target genes.

### Comparison of the efficiency of ChIP using *Arabidopsis* mesophyll protoplasts and transgenic *Arabidopsis* plants

**Fig. 4:**
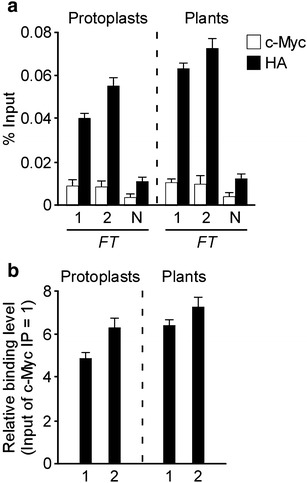
Comparison of SVP binding to the *FT* genomic region in *Arabidopsis* mesophyll protoplasts and transgenic plants. **a** ChIP-qPCR analysis of SVP binding to the *FT* region. The amplified regions within *FT* used for qPCR experiments are shown in Fig. 2a. *35S::SVP:HA* plants used in this study were previously described [18]. The ChIP results obtained from 3 independent biological replicates are represented as percentage of input (% input). *Error bars* indicate the SEM. **b** Relative binding of SVP binding to the *FT* region in protoplasts and transgenic plants. For two amplified regions (1 and 2 in Fig. 2a), the levels of immunoprecipitation by anti-HA antibody were normalized to those of immunoprecipitation by anti-cMyc antibody

We have used both *Arabidopsis* mesophyll protoplasts and *Arabidopsis* transgenic plants to show that SVP binds to the *FT* genomic regions [17, 18]. To compare the efficiency of ChIP using *Arabidopsis* mesophyll protoplasts and *Arabidopsis* transgenic plants, we investigated the degree of binding of SVP to its target motifs within the *FT* genomic region from wild-type mesophyll protoplasts transfected with *35S::SVP:HA* constructs and from *35S::SVP:HA* transgenic plants [18]. We used 2 × 10^7^ protoplasts for transfection of *35S::SVP:HA* constructs and 1 g of plant tissue of *35S::SVP:HA* transgenic plants for this ChIP experiment. ChIP-qPCR analysis showed that binding of SVP transcription factor in the genomic regions of *FT* in mesophyll protoplasts transfected with *35S::SVP:HA* constructs was comparable to that seen in *35S::SVP:HA* transgenic plants (Fig. 4a). Importantly, relative binding of SVP to the *FT* sequences in these two analyses was similar, although the relative binding values from protoplasts were slightly lower than those from transgenic plants (Fig. 4b). Furthermore, we observed approximately five to sixfold more binding compared with a negative immunoprecipitation (IP) control (c-Myc Ab) in protoplasts, which is similar to that observed in transgenic plants (approximately six to sevenfold binding), suggesting that high-quality ChIP-qPCR data can be also obtained from mesophyll protoplasts. These data indicate that our ChIP method using *Arabidopsis* mesophyll protoplasts is as efficient as using transgenic plants, suggesting that a ChIP assay using protoplasts can be a good alternative to a ChIP assay using intact plants.

### The effect of mutation of a binding partner within a protein complex on protein–protein interactions

**Fig. 5:**
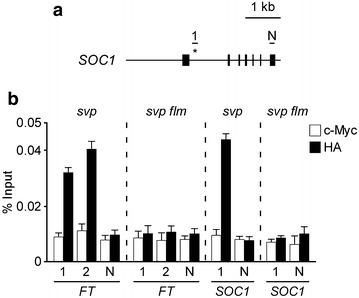
ChIP-qPCR analysis of SVP binding to the *FT* and *SOC1* genomic regions in *Arabidopsis svp* and *svp flm* protoplasts. **a** Diagram of the *SOC1* genomic regions. *Closed boxes* represent exons. The known binding site of SVP [1 (+206 to +454)] in *SOC1* is shown [56, 57]. N, a negative control (+2270 to +2508 in *SOC1*). The amplified regions within *FT* used for qPCR experiments are shown in Fig. 2a. **b** ChIP-qPCR results that show SVP binding to the genomic regions of *FT* and *SOC1* in *svp*-*32* and *svp*-*32 flm*-*3* protoplasts. The ChIP results obtained from 3 independent biological replicates are represented as percentage of input (% input). *Error bars* indicate the SEM

Many transcription factors regulate the expression of their target genes by forming protein complexes with other transcription factors [46–51]. For example, SVP requires the activity of FLOWERING LOCUS M (FLM) to repress the transcription of *FT* and *SUPPRESSOR OF OVEREXPRESSION OF CONSTANS1* (*SOC1*) [18, 52]. To determine whether ChIP-qPCR analysis using mesophyll protoplasts would be suitable to test the effect of a mutation in an interacting protein on binding of the partner to the target sequence, we examined the effect of *flm* mutation on SVP binding to the genomic regions of *FT* and *SOC1* using protoplasts from *svp*-*32* and *svp*-*32 flm*-*3* mutants (both mutants are in the Col-0 background) and transfecting these protoplasts with the *pSVP::SVP:HA* construct. Our ChIP-qPCR analyses showed that strong binding of the SVP transcription factor in the genomic regions of *FT* and *SOC1* was observed in mesophyll protoplasts of *svp*-*32* mutants, whereas the binding of SVP to its target genes in mesophyll protoplasts of *svp*-*32 flm*-*3* mutants was almost abolished (Fig. 5). This result was consistent with the previous finding using *pSVP::SVP:HA svp*-*32* and *pSVP::SVP:HA svp*-*32 flm*-*3* transgenic plants [18]. However, the fold-change of relative binding of SVP to its targets obtained from mutant protoplasts was relatively low, compared to that from complemented transgenic plants [18]. This might be caused by the status of mesophyll protoplasts isolated from pale-green leaves of the mutants [17, 18]. Our data suggest that our ChIP method using *Arabidopsis* mesophyll protoplasts can be also used to test whether binding of a transcription factor to its target sequences requires the formation of transcription factor complexes.

### ChIP-qPCR to test the effect of different temperatures on the binding of a transcription factor to its target sequence

**Fig. 6:**
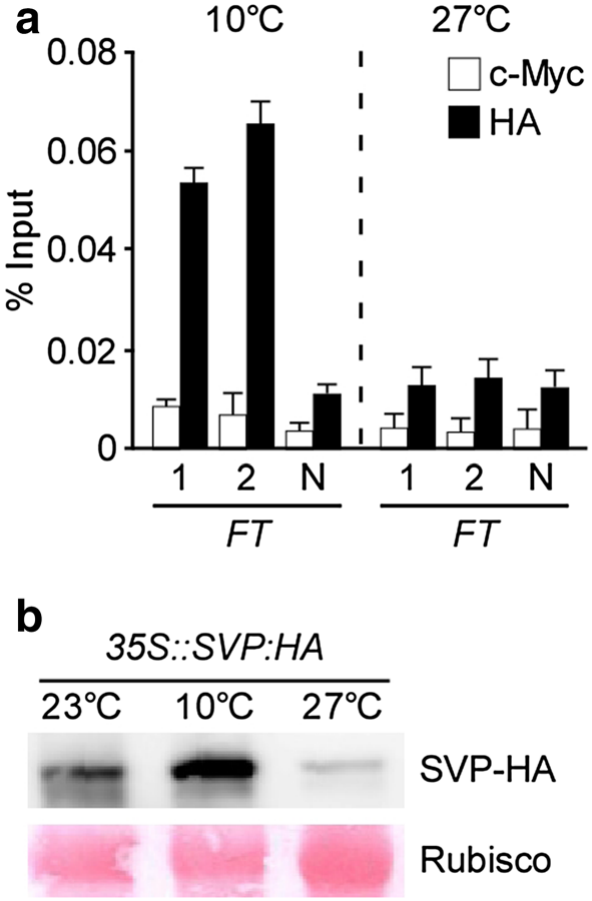
ChIP analysis of SVP binding to the genomic region of *FT* in *Arabidopsis* mesophyll protoplasts incubated at 10 and 27 °C. **a** ChIP-qPCR assay of binding of SVP to the genomic regions of *FT* in *Arabidopsis* mesophyll protoplasts incubated at the indicated temperatures. The *svp*-*32* protoplasts transfected with *35S::SVP:HA* constructs were incubated at 23 °C for 2 h, and then transferred to 10 and 27 °C for overnight. The amplified regions 1, 2, and N (negative control) within *FT* used for qPCR experiments are shown in Fig. 2a. The ChIP results obtained from 3 independent biological replicates are represented as percentage of input (% Input). *Error bars* indicate the SEM. **b** SVP-HA protein expression in *Arabidopsis* protoplasts at different temperatures. Protoplasts were harvested before transfer (23 °C) and after transfer to 10and 27 °C. Western blot analysis was performed using anti-HA antibody to detect SVP-HA protein

Mesophyll protoplasts isolated from leaves can respond to diverse external stimuli such as hormones, metabolites, and pathogens, similar to the responses shown in leaves of whole plants [28–30]. To investigate the effect of temperature on the binding of transcription factors to their targets in *Arabidopsis* mesophyll protoplasts, we compared the binding efficiency of SVP to its target motifs within the *FT* genomic region in mesophyll protoplasts incubated at different temperatures. *35S::SVP:HA* constructs were transfected into protoplasts isolated from *svp*-*32* mutants and then incubated at 23 °C for 2 h. They were subsequently transferred to 10 and 27 °C and incubated overnight. ChIP-qPCR analysis showed that the binding of SVP to the genomic regions of *FT* was observed in mesophyll protoplasts incubated at 10 °C, but not at 27 °C (Fig. 6a). Consistent with the reduction, western blot analysis revealed that the expression of SVP-HA proteins that occurred in protoplasts at 23 °C dramatically decreased in protoplasts at 27 °C but increased at 10 °C (Fig. 6b, Additional file 1: FigureS1), explaining why SVP binding was dramatically diminished at 27 °C. These results suggest that reduced SVP-HA protein levels at a high temperature affect the binding of SVP to *FT* genomic regions, which is supported by previous findings [18]. These data suggest that our ChIP method using *Arabidopsis* mesophyll protoplasts can be used to study the effect of temperature on the binding of a specific transcription factor to its target sequences.

## Discussion

Transcriptional regulation by transcription factors is an initial, critical step to translate genome-encoded information into biological phenomena in living organisms. ChIP assays coupled with qPCR (ChIP-qPCR) and genome-wide analyses such as microarray (ChIP-chip) or deep sequencing (ChIP-seq) provide important insights into the organization and complexity underlying transcriptional regulation by transcription factors. However, conventional ChIP assays using transgenic plants hinder the routine application of this method due to the difficulties in generating transgenic plants that stably express tagged proteins of interest. Here, we describe a fast and efficient ChIP procedure using transient expression in *Arabidopsis* mesophyll protoplasts (Fig. 1).

Several reports have suggested that the optimal concentrations of DNA and the optimal numbers of protoplasts to be used for transfection vary depending on the experimental needs [21]. For example, the quantity of transfected DNA (5–10 kb in size) required for a GFP assay is 10–20 µg for *Arabidopsis* protoplasts [29]. Approximately 1 × 10^3^ to 1 × 10^4^ protoplasts are sufficient for reporter enzyme assays [28] and approximately 1 × 10^6^ protoplasts for microarray analyses [53]. In this study, we observed stronger binding in ChIP-qPCR assays using *Arabidopsis* mesophyll protoplasts when we used 40 µg of DNA and 2 × 10^7^ protoplasts (Fig. 2), compared with assays using less DNA and fewer protoplasts. Furthermore, we successfully detected the binding of tissue-specific transcription factors to their target sequences in *Arabidopsis* protoplasts (Fig. 3), which is comparable to the results obtained using transgenic *Arabidopsis* plants (Fig. 4). However, our suggested conditions may not work universally, as the optimal conditions for efficient transfection of protoplasts may vary with different types of DNA and protoplasts. Thus, the quantity or quality of DNA for transfection and the number of protoplasts should be systematically investigated to identify the optimal conditions for the ChIP assay if one uses our ChIP method in species other than *Arabidopsis*.

A particularly interesting observation in our ChIP-qPCR assay was that *flm* mutation led to almost complete abolishment of SVP binding to the genomic regions of *FT* and *SOC1* (Fig. 5). This suggests that our ChIP method is useful for studying the effect of a mutation in a component of a transcription factor complex on the binding to its target sequences. Another interesting observation was that SVP binding to the genomic region of *FT* decreased in *Arabidopsis* protoplasts at a high temperature (Fig. 6), which was consistent with the previous finding that SVP protein is degraded at high temperatures [18]. These observations suggest that our ChIP method using protoplasts is a good alternative to investigate the effect of different environmental treatments and the effect of a mutation on the binding of transcription factors to their target sequences.

Our ChIP method using mesophyll protoplast has several advantages compared with ChIP assays using transgenic plants. First, our method is time-efficient, such that 4 days in total are required to detect the enrichment of a tagged transcription factor(s) once one decides to test binding of a transcription factor to its target sequence(s). This is particularly important considering that the suitability of transgenic plants that were generated after a long lag time (usually at least several months) for a ChIP assay cannot be guaranteed. In addition, as the suitability of a tag and antibodies to detect the tag can be tested easily and quickly in protoplasts, one can select an optimal combination of tag and antibodies for each experiment. Second, our method is particularly useful to investigate the binding of protein to its target sequence in species or varieties for which transgenic plants are hard to generate, such as crop plants and other economically important plants. Third, our method can bypass some technical difficulties caused by the complexity of plant tissues (i.e., the number of cells in which the transcription factor of interest is active) and other properties of plant tissues (i.e., rigid cell walls, high levels of secondary compounds, and large vacuoles in cells) to prepare samples.

However, our ChIP method using *Arabidopsis* mesophyll protoplasts still has some limitations. First, since the binding of only one transcription factor can be examined at a time, it is not suitable for determining cooperative binding by multiple transcription factors across multiple conditions or multiple time points. Second, when a transcription factor requires a tissue-specific cofactor(s) to bind to its target sequence(s), our ChIP method using mesophyll protoplasts may not be appropriate.

As our method gave high signal-to-noise ratio (i.e., the level of specific binding of transcription factor-bound genomic regions over non-specifically precipitated DNA) in the ChIP experiment (Fig. 4), we propose that our ChIP method can be easily applicable to plants such as rice, maize, and *Brachypodium distachyon* [20, 54, 55], for the analysis of gene regulatory networks in these species for comparative studies of developmental processes such as flowering time, organ development, and translational studies. However, some modifications may be needed for ChIP assays in species other than *Arabidopsis*. We recommend trying our conditions first in species other than *Arabidopsis* and if the ChIP results are not satisfactory, we recommend conducting further species-specific optimization (for instance, protoplast isolation methods, the quantity of DNA and the number of protoplasts used for transfection, and chromatin extraction and sonication).

## Conclusions

In this study, we present a simple, fast ChIP procedure using transient expression in *Arabidopsis* mesophyll protoplasts to study the binding of transcription factors to their target sequences. Our method is easy to perform and involves a minimal amount of handling, equipment, and costs, compared to ChIP assays using transgenic plants. We also show that our ChIP procedure can be used to analyze in vivo interactions between tissue-specific transcription factors and their target sequences, and the effects of a mutation and temperature on the binding of transcription factors to their target sequences. It is of potential interest to any plant scientist who has hesitated to perform ChIP assays due to the lack of appropriate antibodies against a transcription factor of interest and the difficulties in making transgenic plants that stably and highly express transcription factors of interest.

## Additional file

**Additional file 1: Figure S1.** A full image of the western blot shown in Fig. 6b.

## Abbreviations

ARF19: AUXIN RESPONSE FACTOR19
BAT1: BR-RELATED ACYLTRANSFERASE 1
ChIP: chromatin immunoprecipitation
ChIP-qPCR: chromatin immunoprecipitation-quantitative PCR
DTT: Dithiothreitol
EDTA: ethylenediaminetetraacetic acid
EGTA: ethylene glycol-bis(2-aminoethylether)-*N*,*N*,*N*′,*N*′-tetraacetic acid
EMSA: electrophoretic mobility shift assay
FLC: FLOWERING LOCUS C
FLM: FLOWERING LOCUS M
FT: FLOWERING LOCUS T
FUL: FRUITFULL
GL2: GLABRA2
HA: hemagglutinin
LD: long-day
SD: short-day
SEM: standard error of the mean
SEP3: SEPALLATA3
SPL3: SQUAMOSA PROMOTER BINDING PROTEIN-LIKE3
SOC1: SUPPRESSOR OF OVEREXPRESSION OF CONSTANS1
SVP: SHORT VEGETATIVE PHASE
WER: WEREWOLF
