# Supplementary figures and images for: A fast, efficient chromatin immunoprecipitation method for studying protein-DNA binding in Arabidopsis mesophyll protoplasts
Source: Plant Methods. 2017 May 22;13:42. doi: 10.1186/s13007-017-0192-4 (PMC5441002; doi:10.1186/s13007-017-0192-4)

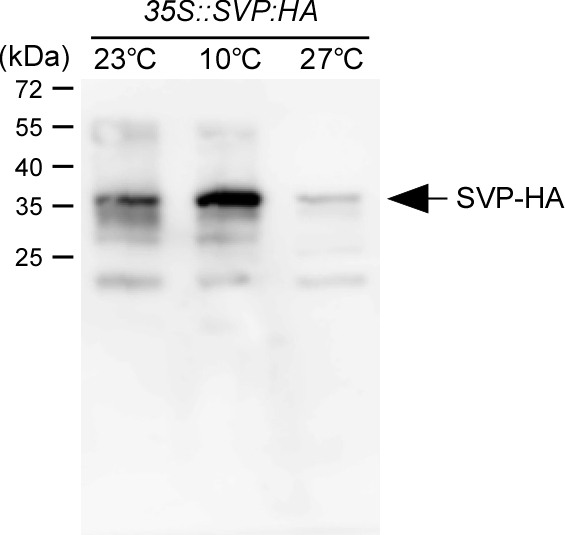


**Figure S1. A full image of the western blot shown in Fig. 6B.**

Supplement: Supplementary file 1 — Additional file 1: Figure S1. A full image of the western blot shown in Fig. 6b. [file 13007_2017_192_MOESM1_ESM.docx]
